# Epidemiological and Molecular Characterization of *Blastocystis* Infection in Children Attending Daycare Centers in Medellín, Colombia

**DOI:** 10.3390/biology10070669

**Published:** 2021-07-16

**Authors:** Maria I. Osorio-Pulgarin, Adriana Higuera, Juan C. Beltran-Álzate, Miryan Sánchez-Jiménez, Juan David Ramírez

**Affiliations:** 1 Instituto Colombiano de Medicina Tropical (ICMT), Universidad CES, Medellín 055450, Colombia; maria.isabel0303@hotmail.com (M.I.O.-P.); jbeltran@ces.edu.co (J.C.B.-Á.); msanchez@ces.edu.co (M.S.-J.); 2 Centro de Investigaciones en Microbiología y Biotecnología-UR (CIMBIUR), Facultad de Ciencias Naturales, Universidad del Rosario, Bogotá 110111, Colombia; adriana.higuera@urosario.edu.co

**Keywords:** *Blastocystis*, protozoa, children, epidemiology, subtypes, diagnosis

## Abstract

**Simple Summary:**

*Blastocystis* infection affects more than 1000 million people globally. Its frequency varies depending on sociodemographic conditions, hygienic habits, provision of public services, and predisposing factors for contracting the infection, considering the child population as the most affected in developing countries. The lack of studies on this protozoan prevents the understanding of its transmission dynamics and its implications for the population’s health. For this protozoan, the diagnosis is based on microscopic reporting by conventional techniques; and subtype identification, which vary depending on the epidemiological distribution, host, and symptoms. In our study, we describe its epidemiological and molecular characterization in a child population. Additionally, we compare the diagnosis methods of *Blastocystis*; our research identified a better sensitivity with molecular methods and a great diversity of alleles and subtypes in this population.

**Abstract:**

Background: The present study aims to perform an epidemiological and molecular characterization of *Blastocystis* infection in a child population attending daycare centers of Medellín, Colombia. Methods: A total of 265 children aged 0–5 years were enrolled in five children’s centers in urban sectors of Medellín, northwestern Colombia. Stool samples were taken to identify intestinal parasites by direct examination, Ritchie–Frick concentration, and molecular identification of *Blastocystis* by conventional PCR and subtype (ST) identification by PCR barcoding with subsequent phylogenetic reconstruction. Kappa index was calculated to evaluate the agreement between microscopy and PCR for the diagnosis of *Blastocystis*. Results: The prevalence of intestinal protozoa was 36.6% (97/265), with *Blastocystis* as the most frequent parasitic protozoan at 15.8% (42/265), followed by *Giardia intestinalis* at 15.5% (41/265) and *Endolimax nana* at 15.1% (40/265). The prevalence of *Blastocystis* by PCR was 53.2% (141/265), the subtypes identified were ST3 at 30.5% (18/59), ST2 at 23.7% (14/59), ST1 at 20.3% (12/59), and with less frequency, ST4 at 5.1% (3/59), ST6 at 1.7% (1/59) and ST16 at 15.3% (9/59) allele 162. Conclusion: This study provides the first genetic characterization of *Blastocystis* subtypes circulating in a population of Medellín, Colombia, and also updates the epidemiology of *Blastocystis* subtypes in the world with the first identification of ST16 in humans.

## 1. Introduction

*Blastocystis* is one of the most frequently isolated intestinal eukaryotes in humans and some animals, such as poultry, dogs, rodents, pigs, reptiles, amphibians, and non-human primates, worldwide [1]. It is currently considered an intestinal protozoan of relevance in public health, as it infects more than a billion of people in the world, and its prevalence is higher in tropical and subtropical areas of developing countries [2,3]. In Colombia, the latest survey carried out by the Ministry of Health reported a prevalence of 52% [4]. Other studies in the country have reported prevalence ranging from 40 to 100% [5,6,7,8]. Despite its high frequency, there is controversy about its pathogenicity. Some authors have found an association with intestinal disorders such as diarrhea, inflammatory bowel disease, irritable bowel syndrome, and extraintestinal manifestations of colitis such as urticaria and iron deficiency anemia [9,10]. Others, on the contrary, find it in equal proportions in symptomatic and asymptomatic patients, generating discussion about its role as a pathogen or commensal [2,11].

At least 17 subtypes represent the genetic diversity of *Blastocystis* (ST), defined by the polymorphisms described in the 18S gene located in the small ribosomal subunit (SSU RNA), with some of them identified in different hosts and others exclusive to humans [12,13]. The epidemiological distribution of STs in humans worldwide is reported in the Americas as ST1, -2, -3, Europe ST1, -2, -3, -4, -5, -6, -7, -8, Africa ST1, -2, -3, -4, -6, -7, Asia ST1, -2, -3, -4, -5, -6, -7, -8, and Oceania ST1, -2, -3, -4, -6, -7, -8, considering ST1, -2, -3 the most frequently found worldwide [14]. Considerable diversity of subtypes has been reported in the United States (14 STs), Brazil (9 STs), and Colombia (8 STs) [15]. Studies carried out in Latin America have shown that humans are mainly infected with ST1, -2, and -3 [16]. In Colombia, several subtyping studies have been carried out in different regions of the country. Therefore, a large part of the diversity of the circulating subtypes present in humans is ST1, -2, -3, -4, -8, -9 [8,16,17], ST4 in non-human primates, ST6 in birds, and ST8 in marsupials [18].

The diagnosis of this protozoan consists of microscopic visualization in stool samples directly, by the concentration, or with stains such as Lugol, Giemsa, or trichrome [19,20,21]. However, the pleomorphism of the parasite hinders adequate morphological visualization [22]. Molecular detection using PCR is more sensitive than microscopy, and enables classification into subtypes [14,23]. Some investigations have reported comparisons between the two tests, microscopy vs. PCR (25.1% vs. 39.2%) [24], (32.3% vs. 88.7%) [25], (33.6% vs. 49%) [8], increasing the prevalence by means of PCR.

The genetic diversity of this protozoan and the possible association with inadequate sociodemographic conditions generate the need of conducting molecular studies in regions of the country where the prevalence is high and not enough studies have been carried out in this regard, as is the case of the department of Antioquia. Specifically, the city of Medellín, which is considered the most populous in the department and the second most populated in the country, with 2.508.452 inhabitants, distributed in 16 communes, mostly from socioeconomic strata of 1, 2, and 3 with access to drinking water in 97% of the population; however, ideal conditions for the transmission of intestinal protozoa can be evidenced, such as inadequate sanitation in vulnerable populations, migrant populations, and displaced persons and victims of conflict, mainly including children. In addition, few studies in the country have compared *Blastocystis* detection by PCR, subtypes, and sociodemographic variables, which is mandatory to unveil the epidemiological distribution of this enigmatic protozoan. Therefore, the objective of this study was to perform an epidemiological and molecular characterization of *Blastocystis* infection in a child population attending daycare centers of the city of Medellín, northwestern Colombia.

## 2. Materials and Methods

### 2.1. Study Population

**Figure 1:**
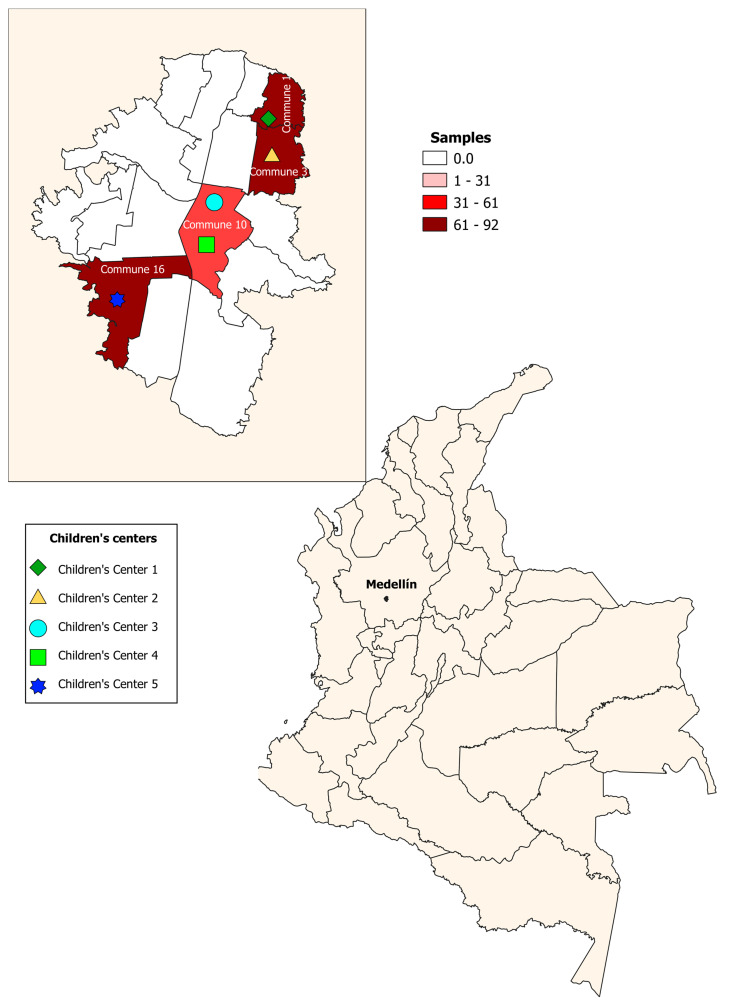
Geographical distribution of children’s centers. Medellín, Colombia.

A cross-sectional study was conducted from 2018–2019. A total of 265 children aged 0–5 years were enrolled in five children’s centers in urban sectors of the city of Medellín; one of the selected centers had sociodemographic conditions of a rural area. These sectors are divided into six zones, and each one of them is subdivided into 16 communes. The children’s centers were located in four communes (communes 1, 3, 10, 16), selected for convenience (Figure 1). Stool samples were taken to identify intestinal parasites by direct examination, Ritchie–Frick concentration, and molecular identification of *Blastocystis* and subtypes. The participants were recruited in the children’s centers, during the monthly meetings of parents or guardians, where the educational and administrative personnel of the institutions were also present. In these meetings, the study was explained, including the methodology and the benefits or contributions to the target population. Informed consent was signed by the parents and guardians to authorize the participation of the children in the study.

### 2.2. Sociodemographic Variables

A survey was carried out that included sociodemographic variables, such as age, sex, nationality, socioeconomic stratum (in Colombia, the strata are from 1 to 6 according to monthly income; strata 1–2 are considered low income, 3–4 middle income, and 5–6 high income), area of origin, children’s center, health affiliation (in Colombia, the health affiliation schemes are subsidized, where the government pays for them, and contributory, where the affiliate and the employer make the payments), diagnosis (identification of intestinal parasites, molecular diagnosis of *Blastocystis*), factors associated with the dwelling (presence of domestic animals, material of the dwelling floor, sanitary service, source of water), and behavioral aspects (habits of importance for intestinal parasites such as playing with dirt, consuming raw or half-cooked meat, and having taken a trip in the last six months).

### 2.3. Parasitological Diagnosis

Using a direct parasitological diagnosis method, two assemblies were carried out, one using saline solution and the other using Lugol stain to visualize the presence of parasites from different families and various compatible developmental stages such as cysts and trophozoites from several parasitic protozoa, as well as eggs of helminths. Additionally, the modified Ritchie–Frick concentration method was used, as suggested by the World Health Organization (WHO) [26].

### 2.4. Molecular Detection of Blastocystis

According to the manufacturer’s instructions, DNA extraction was performed on the 265 samples using the DNA Stool Mini Kit (QIAGEN, Hilden, Germany). For the molecular detection of *Blastocystis*, a PCR was performed in a final volume of 9 µL, containing 3.5 µL of GoTaq Green Master Mix (Promega, Madison, WI, USA), 2 µL of template DNA, and primers FWD F5 (5′-GGTCCGGTGAACACTTTGGATTT-3′) and R F2 (5′-CCTACGGAAACCTTGTTACGACTTCA-3′) as reported elsewhere [8,27].

### 2.5. Subtype Identification and Phylogenetic Analyses

Samples showing positive PCR amplification for *Blastocystis* were subjected to conventional PCR to determine subtypes and alleles, targeting the small subunit of rRNA (ssuRNAr), using the primers RD5 (5′-ATC TGG TTG ATC CTG CCAG T-3′) and BhRDr (5′-GAG CTT TTT AAC TGC AAC AAC G-3′) described above [28]. The PCR products were purified with ExoSap and sequenced on both strands by the Sanger method. The sequences obtained were edited and assembled in Lasergene software version 17.2.1. Subsequently, the sequences, assembled in FASTA format, were submitted to the *Blastocystis* Subtype database (18S), available at http://pubmlst.org/blastocystis/ (accessed on 10 February 2021), and the ST and corresponding alleles were determined through sequence comparison. Phylogenetic reconstruction was performed to observe the clusters of the STs. The consensus sequences of each subtype were aligned with the reference sequences MK719675.1 ST1, MN526751.1 ST2, MK719686.1 ST3, AY244620.1 ST4, AB107964 ST5, AB070990.1 ST6, AB070996 ST7, AB107971 ST8, AF408426 ST9, KC148207 ST10, GU256900 ST11, EU427515 ST12, KC148209 ST13, KC148205.1 ST14, EU427512.1 ST16, and KC148208 ST17 using the multiple sequence alignment program MAFFT v7 (Suita, Osaka, Japan) [29] to construct the tree. A sequence of *Proteromonas* sp. was used as an outgroup to root the final tree. Analysis used 1000 bootstrap replicates in FastTree 2.1 [30]. Each cluster was defined with a bootstrap value of 80.0% and edited using the online tool Interactive Tree Of Life V4 (http://itol.embl.de accessed on 26 June 2021) [31].

### 2.6. Statistical Analysis

The database was prepared in the Microsoft Excel program version 14.0 and reviewed for error control through peer review. Data processing was performed using the SPSS version 22 statistical program. Categorical variables were summarized by relative frequencies and their association with the presence of intestinal parasites (*Blastocystis*, *G. intestinalis*, *Entamoeba coli*, *Endolimax nana*, members of the *Entamoeba* complex, *Iodamoeba butschlii, Chilomastix mesnili*, and *Ascaris lumbricoides*) was assessed using chi-square tests. The odds ratio was calculated with the 95% confidence intervals (CIs) for each of the associations. The bivariate analysis was performed using the Mann–Whitney U test because the data did not have a normal distribution when applying the Kolmogórov–Smirnov test. The kappa index was calculated to evaluate the agreement between microscopy and PCR for the diagnosis of *Blastocystis*. A value close to one indicated that both methods were concordant, and a value close to zero indicated that the methods were not concordant. All analyses were performed in STATA version 14.0 and values of *p* < 0.05 were considered statistically significant.

## 3. Results

### 3.1. Study Population

A total of 265 children aged 0–5 years were enrolled in five children’s centers (center 1 (*n* = 92); center 2 (*n* = 63); center 3 (*n* = 18); center 4 (*n* = 30); center 5 (*n* = 62)). The mean age of the children, ±SD (standard deviation), was 34.1 ± 14.5 months. The age ranged between 6 and 60 months, 89.1% (236/265) of the population was Colombian, and the rest were Venezuelan. It was found that 75.8% resided in urban areas. The most common socioeconomic condition was the low-income stratum (77.3%); the health affiliation scheme was 52.4% subsidized or without affiliation, and the rest were in the contributory regime. Regarding behavioral aspects, it was found that 40.5% of the children play with dirt, 4.9% consume raw or half-cooked meat, and 30.5% had had trips in the last six months.

### 3.2. Infection with Intestinal Parasites

Two hundred sixty-five samples were analyzed by microscopy, finding a frequency of positive samples for intestinal protozoa by the direct method of 30.9% (82/265) and by the concentration method of 32.1% (85/265), for an accumulative prevalence of 36.6% (97/265), with *Blastocystis* infection as the most frequent in 15.8% (42/265) of the samples, as shown in Table 1.

**Figure 2:**
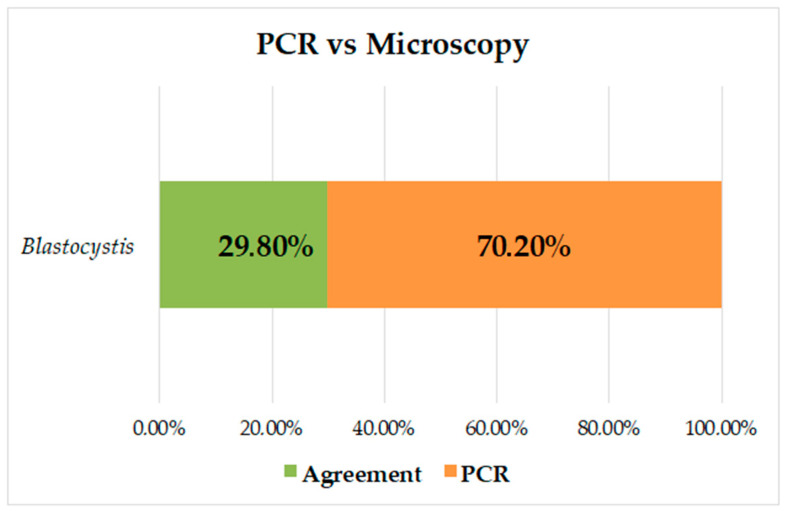
Analysis of concordance between PCR and microscopy measurements for identification of *Blastocystis*.

The prevalence of *Blastocystis* by PCR was 53.2% (141/265), and of the negative samples by microscopy (223/265), 44.4% (99/223) were positive by PCR for *Blastocystis*; there were no samples positive by microscopy and negative by PCR. The kappa index was K = 0.284, and it was found that there was no concordance between the methods; the prevalence was higher when PCR was used for diagnosis, increasing by 37.5% (Figure 2).

### 3.3. Coinfection by Microscopy

**Figure 3:**
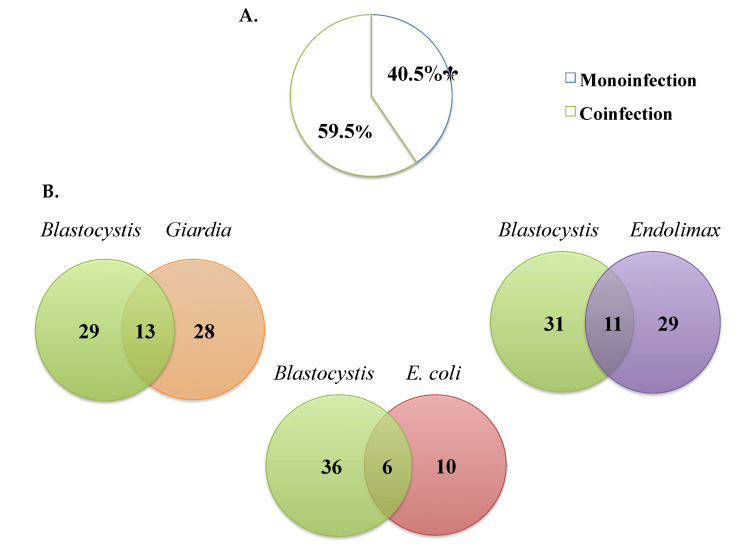
(**A**) Percentages of monoinfection and coinfection in samples positive for *Blastocystis* by microscopy. (**B**) The most frequent coinfections by microscopy.

Of the samples where *Blastocystis* was detected by microscopy, monoinfection was observed in 40.4% (17/42) and coinfection with other parasites in 59.5% (25/42) (Figure 3A). The most frequent *Blastocystis* coinfections were with *Giardia intestinalis* (33.3%), *Endolimax nana* (24.4%), and *Entamoeba coli* (5.7%) and, to a lesser extent, *E. histolytica/dispar/moshkovskii* complex (1.3%), *Ascaris lumbricoides* (1.3%), and *Iodamoeba butschlii* (0.6%) (Figure 3B).

### 3.4. Molecular Characterization of Blastocystis

**Figure 4:**
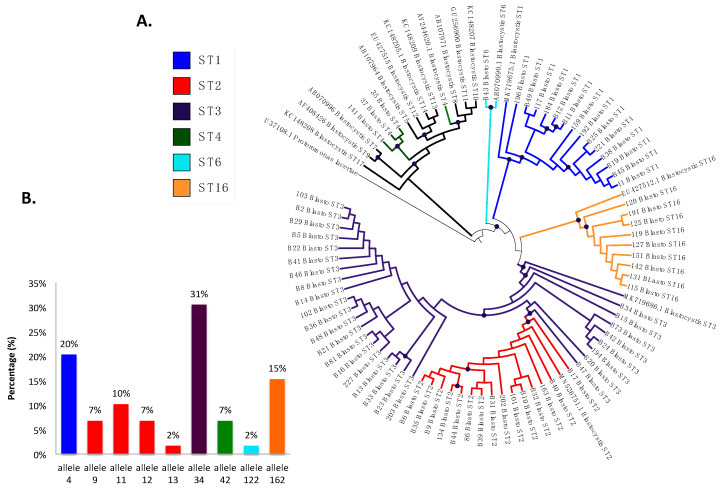
(**A**) Phylogenetic tree of *Blastocystis* subtypes detected in Medellín, Colombia. (**B**) Distribution of *Blastocystis* 18S alleles from the samples analyzed based on each subtype.

The prevalence of *Blastocystis* by PCR was 53.2% (141/265), and 41.8% (59/141) were successfully classified into subtypes (Figure 4A), including ST3 in 30.5% (18/59) of the samples with allele 34, ST2 in 23.7% (14/59), which presented greater allelic diversity, alleles 9, 11, 12, and 13, ST1 in 20.3% (12/59) with allele 4, and, in a lower frequency, ST16 in 15.3% (9/59) with allele 162, ST4 in 5.1% (3/59) with allele 42, and ST6 in 1.7% (1/59) with allele 122 (Figure 4B). In the phylogenetic tree (Figure 4A), well-differentiated clusters of the identified subtypes are observed, some with a bootstrap greater than 80%, which supports the robustness of the phylogenetic reconstruction. We found monophyletic groups of ST1 and ST16 and non-monophyletic ST2 and ST3, with the ST2 cluster bundle within the cluster of ST3. In ST4, the samples bundle in the same cluster but are different from the reference sequence. Sequences were deposited under accession numbers MZ396304-MZ396372. 

### 3.5. Association between Sociodemographic Variables and Intestinal Parasites

Intestinal parasites were found to be associated with age, type of population, children’s centers, health affiliation scheme, and water quality (Table 2). According to age, we found a greater probability of infection at <30 months (OR 0.5 CI 95% 0.346–0.970 *p* = 0.042). According to the type of population, the risk of infection was higher in rural areas (OR 2.2 CI 95% 1.285–4.042 *p* = 0.004) and the probability was also higher in children’s centers in rural areas (OR 3.9 CI 95% 2.267–6.711 *p* = 0.000). According to the health affiliation scheme, it was higher in the subsidized than in the contributory scheme (48% vs. 25%) (OR 2.6 CI 95% 1.551–4.539 *p* = 0.000). Evaluating the dwelling characteristics, we found an association with the water quality, in terms of the frequency of children residing in dwellings having untreated water compared to treated (51% vs. 33%) (OR 0.4 CI 95% 0.20–0.850 *p* = 0.012).

## 4. Discussion

The prevalence of *Blastocystis* infection exceeds 5% in industrialized countries and 30–100% in developing countries [32]. Some studies worldwide report the following prevalence: Iran 14.35% [33], Angola 25% [34], Egypt 55% [10], Thailand 13.6% [35], USA 7% [36], and in Latin American countries: Brazil 40.7% [37], Panama 21.2% [38], and in Colombia, it ranges from 27% to 100% [5,7,8,9,25]. Its frequency varies depending on sociodemographic conditions, hygienic habits and provision of public services, and predisposing factors for contracting the infection, especially in the child population, due to their immunological immaturity, which favors colonization principally by protozoa, becoming a public health problem due to its impact on the morbidity of minors [12,29,30,31]. The prevalence of intestinal parasites in this study was higher with protozoa than helminths. However, a site with rural conditions was selected because it is a marginal area of the city, without drinking water services and with risk factors associated with the transmission of helminthiasis. The other sites were in urban areas, with better socioeconomic conditions, where the risk of infection by helminths could be lower than by protozoa, which could be more associated with food and water contaminated with fecal matter. Garzón et al. states that in Latin America, due to unsanitary conditions, parasites generate a severe public health problem, with greater infection rates of protozoan parasites than helminths [5]. Although some protozoa are not pathogenic for human health, they indicate the ingestion of fecal matter from contaminated food or water and the risk of acquiring other types of parasites that can be considered pathogenic.

A study carried out in Colombia, in a marginal urban area of the city of Medellín and a rural area of the municipality of Unguia-Chocó, compared the diagnosis of intestinal parasites in urban and rural areas. The authors found a similar prevalence in both areas (97.2% and 90%), respectively, with *G. intestinalis* and *Blastocystis* as the most frequent, in agreement with other authors [39,40]. They also found a higher frequency of protozoa than helminths, the latter as the most prevalent in rural areas where poor housing conditions, overcrowding, and inadequate infrastructure for sanitation and access to drinking water are considered factors associated with acquiring the infection [41]. In our study, the protozoa found with the highest frequencies were *Blastocystis* (15.8%) followed by *Giardia intestinalis* (15.5%) and *Endolimax nana* (15.1%) (Table 1). A helminth was also found, *Ascaris lumbricoides*, in four patients, including two of the residents of a site with rural conditions. In another study carried out in Medellín, a prevalence of intestinal parasites of 93% was found, with these parasites considered potentially pathogenic and, among them, *Entamoeba histolytica*/*dispar/moshkovskii* predominated (46.6%), followed by *Giardia intestinalis* (25.9%), *Trichuris trichiura* (25.9%), *Ascaris lumbricoides* (24.1%), and *Enterobius vermicularis* (8.6%) [40]. Although, in the city of Medellín, only the townships are considered rural areas, there are peripheral urban areas with all the characteristics of a rural area with inadequate primary sanitation conditions that allow the transmission of these parasites with higher frequencies than those of protozoa.

One of the reasons that limits the understanding of the pathogenicity of *Blastocystis* infection is the presence of polyparasitism or coinfections where it is not possible to attribute the presentation of symptoms to a single agent when coinfections with agents already considered pathogens occur [17]. The most frequent coinfections by microscopy with *Blastocystis* in our study were with *Giardia intestinalis*, *Endolimax nana*, and *Entamoeba coli* (Figure 3A,B). Ramirez et al. have found, in studies in different regions of Colombia, coinfections with *A. lumbricoides* (16.4%), *T. trichiura* (8.2%), *Enterobius vermicularis* (7.3%), *G. duodenalis* (23.1%), *Entamoeba* complex (82%), *E. coli* (55%), *Hymenolepis nana* (0.8%), *Endolimax nana* (33.2%), and *Neobalantidium coli* (2.7%) by PCR [17]. Another study in Cauca, Colombia, found coinfection by qPCR with *G. duodenalis* and *Cryptosporidium* [24]. A study in Honduras in a child population shows, by multi-parallel real-time quantitative PCR, coinfection with *Trichuris trichiura* [42]. On the other hand, another study in Ecuador found the most frequent coinfections to be *A. lumbricoides* and *G. intestinalis*, without *Blastocystis* as one of the common agents [43]. We did not perform PCR to identify coinfections and, due to the sensitivity of PCR using specific primers or multi-parallel qPCR, coinfections in our study could have been present and could be helpful for future studies. This polyparasitism may be caused by a variable immune response that may be influenced by nutritional status and repeated exposure to various helminths and intestinal protozoa [44]. These findings confirm that intestinal parasite infection continues and generates the need to implement strategies in public health programs establishing control actions regarding access to drinking water, hand washing, and excreta disposal.

Several studies have shown increased sensitivity in the diagnosis of intestinal parasites using molecular methods that, although their cost is high, are effective in making decisions related to control regarding public health with a greater impact [45,46]. For *Blastocystis*, the diagnosis in routine laboratories is based on microscopic reports by conventional techniques [47,48], and only through field investigations are molecular techniques performed to detect this type of intestinal protozoa. In the present study, the prevalence of *Blastocystis* was increased by 37.5% by PCR; all samples positive by microscopy were positive by PCR. However, 44.4% of the samples negative by microscopy were positive by PCR; this percentage and a low concordance between both methods (Figure 2) demonstrate the high sensitivity of molecular diagnosis and draw attention to the possible frequency of false negatives in the diagnosis of this protozoan. Other authors have reported comparisons of microscopy vs. PCR in Panamá (21.2% vs. 74.2%) [38] and in Colombia (25.15 vs. 39.2% [24], 32.3% vs. 88.7% [25], and 33.9% vs. 51.8%) [8]. These results are consistent with what was found in our study and further strengthens the need to use molecular techniques for the diagnosis of *Blastocystis*, showing the increase in false negatives by microscopy, a technique that is more dependent on the observer and, due to its pleomorphism, makes it difficult to visualize it adequately.

Another application for molecular methods is to identify the subtypes of this protozoan, which vary depending on the epidemiological distribution, host, and symptoms [15]. This is the first report of subtyping in Medellín. Some investigations have shown high frequencies of this protozoan, but they have only been of prevalence and epidemiological associations, and molecular studies have not been carried out where the different subtypes are identified [40,41]. In our study, ST1–4, 6, and 16 were found, with ST1–3 as the most frequent (Figure 4), consistent with what was reported in 2019 in an article by Jiménez et al. on the distribution of subtypes in different countries of North and South America (USA, Mexico, Colombia, Brazil, Ecuador, Peru, Bolivia, Chile, and Argentina) [15]. We found ST4 in a small proportion, which coincides with previous reports suggesting that this subtype is of recent origin in humans from the Americas [27] and in Colombia is found in animals such as monkeys (*Alouatta* spp.) and is associated with the enzootic cycle and in a small proportion in humans [8,15].

We also identified, in a small proportion, ST6, and this subtype has been identified with greater frequency in humans in Europe, Africa, and Asia, and is associated with irritable bowel syndrome in patients infected only with *Blastocystis* [14,15]. In Latin America, its distribution has not been very well documented; in Argentina, the prevalence of this subtype is 5%, and in Colombia 2%, where it has been isolated from humans and birds in rural areas of Calarcá and urban areas of Armenia [17]. In a recent study in domestic and captive wild bird species from Brazil, this ST was the most frequent in pheasant and helmeted guineafowl, showing that birds may serve as reservoirs of this subtype and play a role in the transmission to humans [49]. In our study, this subtype was identified in a Colombian child, a resident of an area with rural characteristics, who did not have domestic or farm animals at home at the time of sampling. However, in the houses around the children’s center, there was evidence of the presence of poultry such as chickens that are sometimes in contact with minors, but since these animals could not be included in the study, this statement cannot be confirmed. The possibility remains of studying the subtype dynamics of transmission in the sampled sites and identifying the zoonotic potential, as reported in a study on chickens and humans working in a poultry slaughterhouse in Lebanon where zoonotic transmission was confirmed [50].

Surprisingly, nine samples were positive for ST16, and this is the first report of this subtype in humans in the world; previously, the designation ST16 has been assigned to as yet unpublished sequences from kangaroos obtained by Yoshikawa et al., as part of a survey of marsupials; ST16 also appears to lack a specific related mammalian lineage, with a reptilian *Blastocystis* sequence as its closest relative [51,52,53]. In our study, this subtype was identified in nine children from the same children’s center. It is essential to clarify that this center has sociodemographic conditions of a rural area and the children have contact with rodents, birds, dogs, cats, and pigs that could be infected with intestinal protozoa, increasing the risk of zoonotic transmission, as has been reported in our country and in Brazil [18,54,55]. This report suggests possible zoonotic transmission in the area and opens the possibility of finding new hosts or reservoirs in the *Blastocystis* transmission cycle; for this, it is necessary to continue with subtyping studies that include domestic and farm animals. The question is about the zoonotic role of *Blastocystis* and the epidemiological distribution of its subtypes since, initially, this ST was found in kangaroos and could be phylogenetically related to that of reptiles. However, it is still the subject of research.

We also characterized the alleles of each of the STs (Figure 4B), and allele 34 of ST3 was the most frequently observed, followed by allele 4 of ST1, and the presence of alleles 9, 11, 12, and 13, provided evidence of the intra-subtype diversity present in ST2, as previously reported in studies of STs circulating in Ecuador, Peru, Bolivia, Brazil, Argentina, and Colombia [8,16,24]. For ST4, allele 42 was identified, which had previously been reported in Europe and Colombia [18,55]; allele 122 was identified for ST6, as previously reported, usually as a zoonotic subtype but also as a small proportion in humans [15,16]. For ST16, we detected allele 162, of which there are no previous reports in the world. This suggests again the high intra- and inter-ST diversity evidenced in *Blastocystis* which implies the need to continue unveiling its implications for different characteristics of this infection.

Several authors have found that some sociodemographic characteristics are associated with the risk of acquiring an infection with intestinal parasites [4,34,56]; in a study in Thailand, Leelayoova et al. found that those older than five months are more susceptible to acquiring this type of infection because their habits are more related to inadequate hygiene conditions, which increase exposure to infection [36]. In Colombia, Londoño et al. found that infection is more frequent in those older than 48 months (62.4%) [6]; contrary to this, in our study, we found that those under 30 months present a higher frequency of infection, being statistically significant, as they may have less control over hand washing and are more exposed to contamination in their food, supporting the hypotheses mentioned above about the role of minors in the transmission of intestinal parasites.

In our study, we confirmed that inadequate sociodemographic conditions such as poor water quality, generally in marginal areas with rural characteristics where hygienic habits are not well implemented, are factors associated with parasitic infections, finding statistically significant differences (Table 2), consistent with those reported by other authors in rural and marginal areas of Colombia [39,41]. However, other authors in the same country have not found an association between sociodemographic characteristics and parasitic infections [24]; another study in the indigenous reservation in Nasa, Cauca, did not find statistical associations between sociodemographic conditions and parasitism. However, the authors described the low availability of aqueducts and sewerage systems that may play a role in transmitting these types of infections [56]. Sociodemographic aspects continue to be controversial in terms of the presence of intestinal parasite infections and their proper management shows an increase in frequencies, especially in the child population of these marginal areas in Colombia; it is essential to identify which of these variables could be associated with *Blastocystis* infection. On the other hand, as a limitation of our study, it was not possible to perform this since the infection was only determined by microscopy and, statistically, it was not feasible to attribute the association to a single parasite. For this, it would have been necessary to implement molecular techniques to determine the presence or absence of other species of intestinal parasites.

## 5. Conclusions

This study provides the first genetic characterization of *Blastocystis* subtypes circulating in a population of Medellín, Colombia, and also updates the epidemiology of *Blastocystis* subtypes in the world with the first identification of ST16 in humans. These findings suggests the need to apply molecular markers with higher resolution to describe the transmission dynamics of *Blastocystis*, including a variety of animals, to understand the epidemiology and sources of transmission to humans.

## Figures and Tables

**Table 1:** Prevalence of intestinal parasites according to the diagnostic method used.

Intestinal Protozoa	Diagnostic Method		
	Direct	Concentration	Prevalence
Protozoa			
*Blastocystis*	14.7% (39/265)	11.7% (31/265)	15.8% (42/265)
*Giardia intestinalis*	12.5% (33/265)	15.1% (40/265)	15.5% (41/265)
*Endolimax nana*	10.6% (28/265)	9.15% (24/265)	15.1% (40/265)
*Entamoeba coli*	4.2% (11/265)	5.7% (15/265)	6% (16/265)
*Entamoeba* complex	1.1% (3/265)	0.8% (2/265)	1.1% (3/265)
*Iodamoeba butschlii*	0.8% (2/265)		0.8% (2/265)
*Chilomastix mesnili*	0.8% (2/265)	1.1% (3/265)	1.1% (3/265)
Helminths			
*Ascaris lumbricoides*	0.8% (2/265)	0.8% (2/265)	0.8% (2/265)

**Table 2:** Sociodemographic variables studied under statistical analysis.

Variable	Category	Intestinal Parasites				
		Positive	Negative	Total	OR (CI 95%)	*p*-Value
Sex	Male	42 (31.6%)	91	133	0.6 (0.391–1.069)	0.088
	Female	55 (41.6%)	77	132		
Age	>30 months	88 (58.6%)	62	150	0.5 (0.346–0.970)	0.042
	<30 months	53 (46.1%)	62	115		
Nationality	Venezuelan	13 (52%)	12	25	2.0 (0.926–4.732)	0.108
	Colombian	79 (35.6%)	143	222		
Type of population	Rural	33 (51.6%)	31	64	2.2 (1.285–4.042)	0.004
	Urban	64 (31.8%)	137	201		
Children’s center	2 *	33 (52.3%)	30	63	3.9 (2.267–6.711)	0.000
	1, 3, 4, 5 **	64 (31.6%)	138	202		
Health affiliation scheme	Subsidized	61 (48%)	66	127	2.6 (1.551–4.539)	0.000
	Contributory	31 (25.8%)	89	120		
Stratum	1-2	73 (38.2%)	118	191	1.2 (0.645–2.252)	0.559
	3-4-5	19 (33.9%)	39	56		
Floor material	Land	2 (50%)	2	4	0.5 (0.81–4.24)	0.595
	Cement tile	90 (37%)	153	243		
Water quality	Treated	64 (33.2%)	129	193	0.4 (0.20–0.850)	0.012
	Untreated	28 (51.9%)	26	54		
Pets	Yes	31 (37.8%)	51	82	1.0 (0.600–1.79)	0.898
	No	61 (37%)	104	165		

*p*-value: <0.05, OR: odds ratio. * Children’s center with sociodemographic conditions of a rural area. ** Children’s centers in urban areas.

## Data Availability

All data generated or analyzed during this study are included in this published article. Sequences were deposited under accession numbers MZ396304–MZ396372.
